# On the preconditions for large-scale collective action

**DOI:** 10.1007/s13280-019-01284-w

**Published:** 2019-11-12

**Authors:** Sverker C. Jagers, Niklas Harring, Åsa Löfgren, Martin Sjöstedt, Francisco Alpizar, Bengt Brülde, David Langlet, Andreas Nilsson, Bethanie Carney Almroth, Sam Dupont, Will Steffen

**Affiliations:** 1 Department of Political Science, University of Gothenburg, Box 711, Sprängkullsgatan 19, 405 30 Gothenburg, Sweden; 2 Department of Economics, University of Gothenburg, Box 650, 40530 Gothenburg, Sweden; 3 Environment for Development Initiative, CATIE, Turrialba, Costa Rica; 4 Department of Philosophy, Linguistics and Theory of Science, University of Gothenburg, Box 200, Olof Wijksgatan 6, 41255 Gothenburg, Sweden; 5 Department of Law, University of Gothenburg, Box 650, 40530 Gothenburg, Sweden; 6 Department of Psychology, University of Gothenburg, Haraldsgatan 1, 405 30 Gothenburg, Sweden; 7 Department of Biological and Environmental Sciences, University of Gothenburg, Box 463, Medicinaregatan 18, 405 30 Gothenburg, Sweden; 8 Fenner School of Environment & Society, The Australian National University, Building 141, Linnaeus Way, Canberra, ACT 2601 Australia; 9 Department of Social Sciences, Wageningen University and Research, P.O. Box 8130, 6700 EW Wageningen, The Netherlands; 10 The Kristineberg Marine Research and Innovation Centre, University of Gothenburg, 566 Kristineberg, 45178 Fiskebäckskil, Sweden

**Keywords:** Facilitators, Global commons, Large-scale collective action, Social dilemmas, Stressors

## Abstract

The phenomenon of collective action and the origin of collective action problems have been extensively and systematically studied in the social sciences. Yet, while we have substantial knowledge about the factors promoting collective action at the local level, we know far less about how these insights travel to large-scale collective action problems. Such problems, however, are at the heart of humanity’s most pressing challenges, including climate change, large-scale natural resource depletion, biodiversity loss, nuclear proliferation, antibiotic resistance due to overconsumption of antibiotics, and pollution. In this paper, we suggest an analytical framework that captures the theoretical understanding of preconditions for large-scale collective action. This analytical framework aims at supporting future empirical analyses of how to cope with and overcome larger-scale collective action problems. More specifically, we (i) define and describe the main characteristics of a large-scale collective action problem and (ii) explain why voluntary and, in particular, *spontaneous* large-scale collective action among individual actors becomes more improbable as the collective action problem becomes larger, thus demanding interventions by an external authority (a third party) for such action to be generated. Based on this, we (iii) outline an analytical framework that illustrates the connection between third-party interventions and large-scale collective action. We conclude by suggesting avenues for future research.

## Introduction

The phenomenon of collective action and the origin of collective action problems have been extensively and systematically studied in the social sciences. A collective action problem is normally described as a situation in which the short-term self-interest of individual actors is in conflict with longer-term collective interests, generating a substantial risk that the collective benefit is not produced at all (Olson 1965). For example, the late Nobel laureate Elinor Ostrom (Ostrom 1990, 2000, 2005, 2011) showed that local users of a common resource can overcome collective action problems by setting up systems of self-governance among resource users in, for example, a fishing village or a farming community (Bromley 1992; Agrawal and Gibson 1999, 2001). Yet, while we have substantial knowledge about the factors promoting collective action at the local level (Ostrom 1990; Agrawal 2001), we know far less about how these insights transfer to large-scale collective action problems and their solutions, including climate change, large-scale natural resource depletion, nuclear proliferation, antibiotic resistance, and pollution. In particular, while successful large-scale collective action has occurred both nationally, such as tax collection and public goods provisioning in welfare states (Rothstein 2001), and internationally, in terms of successful environmental agreements such as the Montreal Protocol on substances that deplete the ozone layer, there has been little systematic theorising on the prospects for large-scale collective action in general.

Accordingly, rather than conduct an exhaustive literature review or an empirical investigation, we instead suggest in this paper an analytical framework that captures the theoretical understanding of preconditions for large-scale collective action. This analytical framework attempts to support any future empirical analysis into coping with and overcoming larger-scale collective action problems. We thereby set the stage for future research and the development of necessary policies to reach crucial targets such as the UN Sustainable Development Goals. By doing so, this paper also serves important pedagogical functions: by deriving our analytical framework, we provide a distinct explanation as to why making actors aware of their environmentally detrimental behaviour, such as through information campaigns, is seldom sufficient to induce behavioural changes. Our approach also provides an explanation as to why even very strong pro-environmental values, norms, and beliefs should not be expected to result in any substantial behavioural changes among involved actors (other than under exceptional conditions). Most of these transformations require active assistance from one or many external parties.

More specifically, our objectives are threefold. First, based on the comprehensive existing literature on collective action, we define and describe the main characteristics of a large-scale collective action problem. Second, we explain why voluntary, and especially *spontaneous*, large-scale collective action becomes more improbable as the collective action problem becomes larger, thus demanding interventions by external authorities (“third parties”). Third, and most importantly, we outline an analytical framework capturing the connection between third-party interventions and large-scale collective action.

In Section “The logic of collective action and social dilemmas”, we define collective action and then briefly review the most prominent factors generating successful collective action. We refer to these factors as collective action *facilitators*. In Section “The logic of large-scale collective action”, we first specify the concept of large-scale collective action and then identify the main *characteristics* of large-scale collective action problems. Thereafter, we explain how these characteristics generate *stressors* that make successful large-scale collective action less likely. We conclude that large-scale collective action needs to be supported by various types of interventions carried out by a third party, such as the state, a trade association, or a social movement. The question of when and how such third parties can be created or evolve endogenously—and intervene with sufficient legitimacy and effectiveness—is, in turn, an issue for future research to explore. In Section “Discussion: the dynamics of a large-scale collective action problems and third-party interventions”, we graphically illustrate our analytical framework and discuss its implications for human cooperation. Section “Concluding remarks” concludes with a summary of our argument, an application of the proposed framework, and discussion of potential paths for future research.

## The logic of collective action and social dilemmas

Problems of *collective action* permeate societies on all levels, from the very local to the global, and they cross both political borders and generations (Ostrom 1998). A collective action problem is typically described as a situation in which actors are motivated to take a course of action that is more beneficial than costly to them individually but is more costly than beneficial to society. This generates a substantial risk that collective benefits will not be produced. In the social science literature, a collective action problem is typically understood as a social dilemma. Building on Dawes’s seminal definition (Dawes 1980, p. 170), a social dilemma is present when both of the following premises are true:

1. The payoff for each individual actor to act in self-interest (called *defecting*) is higher than the payoff for acting in the interest of the collective (called *cooperating*), regardless of what others do.
2. All individual actors receive a lower payoff if all defect than if all cooperate.

Using social dilemma logic is a powerful way of explaining the origin of environmental problems and discussing how they can be overcome. Perhaps the most prominent example is Hardin (1968), who, inspired by the British economist William Forster Lloyd, introduced the idea of the “tragedy of the commons”. This is when individual users in a shared resource system, acting independently according to their own short-term self-interest, behave contrarily to the common good of all users by depleting or spoiling the shared resource through their collective action.

However, not all collective action problems, especially larger-scale collective action, are proper social dilemmas. Other coordination problems should be included as well, such as (1) situations that do not necessarily affect an individual actor, but rather affect other parties, such as patients, children, clients, or future generations, and thus also (2) situations where a principal, representing or captaining a group of actors, must come to an agreement with other such principals in order to eventually achieve behavioural changes among these actors, ultimately causing the problem through their individual defecting behaviour. In addition, collective action problems also include “race-to-the-bottom” situations, where even a small number of defecting actors can start a negative feedback loop, making cooperation less likely.

Therefore, all types of collective action problems share a feature of proper social dilemmas: they cannot be overcome, or managed, unless at least *some actors act against their own short*-*term self*-*interest, or against the interest of their principals* (i.e. cooperate rather than defect). A complete analytical framework of collective action should cover all these types of collective action problems.

### Collective action facilitators

The phenomenon of collective action has received a tremendous amount of scholarly attention, primarily in the form of laboratory experiments or local field studies of self-regulating regimes. These studies of voluntary and spontaneous collective action on a smaller scale have resulted in findings that today are seen as core facts, contradicting the zero-contribution (defection) hypothesis dictating that all actors are rational egoists (Ostrom 2000). For example, there is ample empirical support for assuming that the vast majority of people are at least *conditional* cooperators in collective action situations—that is, they are willing to cooperate given certain premises, such as whether other actors are cooperating too (Fischbacher et al. 2001; Gächter and Herrmann 2009).

A large number of factors, which we term collective action *facilitators*, have been shown to determine the conditions for, and affect the prospects of, cooperative behaviour in numerous smaller-scale cases. Though not an exhaustive review, here we note some interesting and illuminating examples. People tend to exhibit a willingness to *accept costs in order to punish free riders* (Fehr and Gächter 2000a), and *punishment,* or the threat of punishment, can also reduce the incidence of free riding and sustain high levels of cooperation (Gächter and Herrmann 2009). The level of cooperation (e.g. in public good experiments) is in turn affected by whether the experiments are *anonymous* or *public* (Laury et al. 1995). Furthermore, individuals tend to increase their contributions if each person’s contribution is publicly disclosed (Gächter and Fehr 1999). Thus, people seem to be willing to cooperate if their reputation is at stake. In addition, levels of cooperation in laboratory collective action-type games substantially increase if the subjects have the possibility of communicating with each other (Sally 1995). Cooperation is also affected by the characteristics of the collective good (Dietz et al. 2002). Likewise, the influence of group size on levels of cooperation has also been repeatedly studied and disputed (Isaac et al. 1994; Agrawal and Goyal 2001; Carpenter 2007). On a related note, Ostrom found that the links among trust, reciprocity, and reputation are at the core of behavioural explanations of cooperation in collective action dilemmas (Ostrom 1990, 1998, 2005). In situations with high levels of initial cooperation, more individuals tend to adopt reciprocity as a norm, and if reciprocity is widespread, having the reputation of being trustworthy becomes a good investment. This implies that levels of trust in other people, norms of reciprocity, and having a reputation for being trustworthy are more or less mutually reinforcing. Given how difficult it is to overcome collective action dilemmas, however, the opposite direction of causality may be present too. That is, reciprocity can also be *detrimental* to cooperation. The best example of this is when an actor, based on past experiences, expects that others will not cooperate and therefore chooses to not cooperate (Fehr and Gächter 1998).

The facilitators listed are often interconnected and reinforce and facilitate each other. For example, punishment can be a way to foster and maintain social norms, and technological solutions can be a way to facilitate communication.

## The logic of large-scale collective action

Theories about collective action claim and show that simple exchanges often can be governed—and coordination and cooperation problems overcome—by mechanisms such as trust, reciprocity, and reputation. Thus, if two actors are involved in repeated interactions, or if the actions of one of the actors can easily be monitored by the other actor, the risk of reneging decreases substantially (Ostrom and Walker 2003). However, such bilateral mechanisms become less efficient in large-scale cooperation or coordination problems. This is because, with larger problems, it is less likely that the actors involved will be able to coordinate themselves. Most important, they cannot directly monitor the performance or anticipate the actions and outcomes of other actors. This creates a demand for a third party with the capacity to reduce uncertainty by providing cognitive, coordinative, normative, and informational guidance (Greif 2006).

While scale might appear to be an evident factor obstructing cooperative behaviour, current literature on collective action has, however, failed to adequately address the different characteristics that generate or hamper larger-scale collective action. Instead, most evidence on the prospects for collective action still stems from small-N experiments and single case studies. These types of studies are not particularly relevant, nor are they representative of many of the present challenges that humanity is facing, including climate change, ocean acidification, biodiversity loss, pollution, antibiotic resistance, or the achievement of many of the other UN Sustainable Development Goals. What we do see, however, is a growing body of research primarily addressing the complexity of global challenges (see Young 2002; Steffen et al. 2006; Biermann 2007; Scheffer 2009; Steffen et al. 2011; Biermann 2012; Galaz et al. 2012; Steffen et al. 2015, Berkes 2017; Young 2017).

As we see it, the scholarly community needs to recognise and understand these global challenges as collective action problems, and more specifically, as collective action problems in the broader sense advocated in Section “The logic of collective action and social dilemmas”. In particular, not enough attention has been paid to the characteristics affecting large-scale collective action and thus, by extension, to the question of how to generate and sustain collective action in respect to these challenges. Therefore, we refer to Ostrom’s later work, in which she was becoming gradually more concerned with polycentric systems and cross-system interactions, such as in her *institutional analysis and development framework* (Ostrom 2005, 2010). To build on this work, however, we need to more thoroughly discuss what characterises a *large*-*scale* collective action problem.

These are important endeavours, because without properly understanding the precise nature of each large-scale collective action problem, and analysing it accordingly, not only is it impossible to identify the fundamental causes and mechanisms behind each problem but it is also impossible to find successful ways and policy instruments to overcome them.

### Characteristics of large-scale collective action problems

One way to characterise a large-scale collective action problem is to assess the impact or magnitude of the problem at hand. For example, the loss of a local fish stock is typically considered a smaller-scale problem than the loss of global fish stocks, and pollution in a pond is a smaller-scale problem than ocean pollution. Those examples also constitute good illustrations of the difference in resource characteristics between small(er) and large(r)-scale collective action problems, where, for example, larger-scale resources are often migratory rather than stationary, which affects the potential for cooperation and sustainable use (Ostrom 1990). However, to fully understand prospects for large-scale collective action in relation to large-scale problems, one has to identify the defining characteristics constituting large-scale collective action problems and the mechanisms (stressors) producing these undesirable impacts. Before we introduce these characteristics and stressors, it is important to state that we see no clear borders beyond which a collective action problem becomes large scale. Instead, collective action problems are better described on a continuum from smaller to larger scale.

#### Number of actors

The number of actors involved is probably the most apparent characteristic of a large-scale problem. While group size has been studied earlier with ambiguous effect (Messick 1973; Isaac et al. 1994; Carpenter 2007), increasing the number of actors on a larger scale reduces the likelihood of collective action for at least two reasons. First, the more actors involved, the more difficult coordination and cooperation become. Hence, the collective usage is more likely to have a significant negative impact on the resource or collective good in question. Second, to facilitate coordination among a large number of actors, representatives are often introduced. However, such representatives may act in their own self-interest rather than on behalf of their principals, such as their children, patients, voters, clients, or shareholders (Adserà et al. 2003). This is very different from, for example, a communal irrigation system in which each farmer can represent himself or herself. Hence, large-scale collective action problems are often characterised by the presence of representatives and consequently involve agency problems, such as corruption and problems related to monitoring and surveillance (Milgrom et al. 1990; Greif et al. 1994).

#### Spatial distance

An additional characteristic of many large-scale problems is that they affect large geographic territories that span *multiple countries* (as in the case of acid rain), *multiple continents* (as in the case of overfishing), or the *whole world* (as in the case of climate change or antibiotic resistance). Sometimes this is due to massive detrimental activities more or less evenly distributed across the globe (e.g. greenhouse gases), resulting in the global spread of the problem. However, sometimes even relatively few local sources can still have a very large-scale and widespread impact (e.g. a damaged nuclear reactor or local pollution that is distributed via the atmosphere, the oceans, or rivers). It is important to recognise that the geographic distribution of a large-scale problem is always directly related to the number of actors either affected by or causing the problem.

#### Temporal distance

The time lag between the causes (actions of individual actors) and the aggregated effects strongly influences the likelihood of the emergence of large-scale collective action (Milfont et al. 2012; Hauser et al. 2014). Many of the larger-scale challenges that we see today have a comparatively long temporal distance. One example is societies’ use of substances generating waste that typically lasts for generations. An extreme example is nuclear waste, since many radioactive isotopes have half-lives of tens to hundreds of thousands of years. Another area where the temporal distance is long is climate change, as some greenhouse gases will have an active impact on global warming for several hundred years, and sea levels can continue to rise over thousands of years.

Once again, the interaction between these characteristics needs to be recognised. A concern about long-lasting pollutants immediately opens up a discussion of multiple generations being affected, thereby once again dramatically expanding the number of actors relevant to and dependent on the solution.

#### Complexity

Large-scale collective problems are also typically characterised by a large degree of complexity, which can result in a reduced understanding of the problem and an inability to comprehend and perceive its consequences. For example, unlike most small-scale collective problems and dilemmas, with large-scale problems, boundaries are unclear, the evidence is patchy, and the scientific underpinning of both the problem and the solution is often debatable. For example, in an inshore fishery, fishermen can personally observe that the resource is being overused, whereas understanding overuse of the atmosphere as a depository of greenhouse gases, and the consequences of this overuse, requires extensive, interdisciplinary scientific research and knowledge.

To make matters even more complex, larger-scale collective problems are typically interconnected. For example, carbon dioxide emissions, biodiversity loss, and ocean acidification are all large-scale dilemmas in themselves, but they are also strongly interconnected (Steffen et al. 2018). Another illustrative example is the sea ice–albedo relationship and its connection to climate change. When sea ice melts, a feedback cycle occurs as open water absorbs more sunlight than ice. This leads to further regional warming, which leads to further loss of ice, and so on (Deser et al. 2000; Scott and Hansen 2016). This mechanism contributes to climate change at the planetary level, hence becoming a global problem.

Moreover, large-scale problems can lead to the collapse of small-scale collective agreements, such as when changes in rainfall alter the carrying capacity of an irrigation system, resulting in the failure of old agreements, even if all parties comply with the terms. The level of complexity of large-scale problems is amplified by the fact that multiple regions are affected, sometimes very differently, and also by the need to account for consequences that span many years, decades, or even centuries into the future.

Finally, it should be recalled that the characteristics of the large-scale collective action problems typically differ depending on the nature of the problem. For example, there are great differences in characteristics between marine plastic pollution, where a large number of actors are involved but both the temporal distance and the complexity are rather low, and global climate change, which scores high on all four characteristics. Nonetheless, they are both very large scale.

### Stressors counteracting successful collective action

The four characteristics described above are all *defining* characteristics in the sense that they, either alone or in combination, determine what constitutes a *large*-*scale* collective action problem (or at least *larger*-scale on a continuum ranging from small to very large). Why and how do these characteristics affect actors’ collective action behaviour? Our core argument here is that either in isolation or in combination, these four defining characteristics give rise to a number of stressors that negatively affect the prospects of collective action. These stressors should be understood as mechanisms explaining why larger-scale collective action is less likely to occur. Below we derive what we perceive to be some of the most obvious (and dominating) stressors and give brief descriptions of their impact on the likelihood of collective action as a solution to large-scale problems.

#### Anonymity

With an increasing number of actors involved, it becomes more likely that the actors will be anonymous to each other. Additionally, this anonymity is reinforced as the spatial and temporal distances increase. In the extreme case of a problem spanning multiple generations, anonymity between individuals in different generations, some of them yet to come, is probably absolute. Anonymity is detrimental for cooperation: as the actors become more anonymous, it becomes increasingly difficult to reduce free riding, since actors cannot engage in face-to-face communication, exchange promises, or monitor that promises are being kept (Greif 1993; Ostrom 1998). Anonymity also has negative impacts on collective action facilitators such as the maintenance and communication of jointly held social norms.

#### Lack of accountability

With an increasing number of actors, as well as larger spatial and temporal distances, the possibility of observing individual actions tends to decrease. In addition, each individual’s relative contribution to the collective action problem becomes smaller and harder to single out. The result is a perception that individual actions do not carry an impact and therefore that individuals are not fully accountable for their actions. For example, an individual’s personal contribution to global warming and climate change is infinitesimal. Therefore, it is easy, and psychologically tempting, to whitewash the (shared) responsibility that most individuals might have to lower their impact on the climate system. Furthermore, the global level lacks similar accountability mechanisms (e.g. elections) that can be present at the national level (Grant and Keohane 2005; Duus-Otterström and Jagers 2012).

#### Heterogeneity

Several of the large-scale characteristics tend to generate several forms of heterogeneity, including differences in identities, socioeconomic status and power asymmetries, cultures, traditions, and religions, each of which jeopardises the mechanisms that generate cooperation, not least the levels of trust and perceptions of fairness between actors (Baland and Platteau 1996; Bardhan and Dayton-Johnson 2000; Varughese and Ostrom 2001; Ostrom 2010). Through these various types of heterogeneities, large-scale characteristics such as the number of actors, the temporal distance, and the spatial distance, decrease the potential for establishing and sustaining reciprocal relationships. In the case of temporal distance, reciprocity is even unattainable. This is valid for both positive reciprocity (e.g. services in return) and negative reciprocity (e.g. sanctions).

#### Risk and uncertainty

Large-scale characteristics accentuate uncertainty and risks about consequences, as well as knowledge concerning which actors give rise to these consequences (in both the societal and ecological spheres) (Wit and Wilke 1998). First, there may be environmental uncertainty and risk, such as the actors’ lack of knowledge about the size of a common resource (Messick et al. 1983; Wit and Wilke 1998). Quite often in large-scale problems, there is no or incomplete environmental information, which may result in an unintended pressure on the resource (Messick and McClelland 1983). Second, there may be social uncertainty and risk, such as a lack of knowledge about other actors’ choices and actions. Studies show that when participants are unaware of how others in a group act, they are less cooperative (Rapoport et al. 1992). The negative effects of uncertainty are typically exacerbated when information is not evenly distributed across the spatial dimension or when there is a lack of trust in the institutions that are supposed to provide the information. Furthermore, a lack of information today and the promises of all-encompassing solutions in the future (e.g. promises that climate change can be overcome by geoengineering) might lead to inaction today.

#### Emotional detachment and cognitive limitations

Large and complex problems spanning vast territories and multiple generations constitute a heavy burden on humans’ cognitive abilities. Theories about “Bounded rationality” (Kahneman 2003) implies that human problem solving is constrained by a limited cognitive ability that results partly from the brain’s autonomous decision not to spend too much time and effort on every decision. When actors are confronted with complex large-scale collective action problems, characterised by long spatial and temporal distances, this may generate an emotional detachment that leads to inaction. For example, psychological distance to climate change consequences has been shown to affect the intensity with which emotions are experienced (Van Boven et al. 2010; McDonald et al. 2015). Emotions are generally less intense with increased psychological distance to the emotion-eliciting event. People may also perceive events in different ways related to spatial and temporal distance. As suggested by construal level theory (Trope and Liberman 2010), objects, events, and constructs can be thought of in more or less abstract terms depending on the psychological distance to them. The further away something is perceived to be from one’s immediate experience, the more abstract the construct or event will be perceived. Thus, even when people are informed about, and become aware of, the negative consequences of climate change, for people that are relatively spatially and/or temporally distant, they may not be willing to act on that information because of less emotional intensity or a more abstract construal of the event.

## Discussion: The dynamics of a large-scale collective action problems and third-party interventions

Because of the various stressors originating and previously derived from the large-scale characteristics of the problem, the following premise can be put forward:

The larger the scale of the collective action problem, the less likely it is that the collective action facilitators will be strong enough to outweigh the negative effect of the stressors caused by the large-scale characteristics.

Hence, the theories and findings about collective action (as summarised in Table 1) showing that simple exchanges can often be governed, and coordination and cooperation problems overcome, voluntarily and by informal mechanisms such as trust and reciprocity or by local-level institutional arrangements (Ostrom and Walker 2003), are typically not applicable to many large-scale collective action problems and situations. For example, if an upstream polluter happens to be the most powerful actor along a polluted transnational river, there are few benefits for this polluter to start reducing emissions. Furthermore, it is not very likely that the downstream actors who are suffering from the pollution will be able to persuade or force the upstream polluter to change its behaviour. This collective action situation is clearly different from the social dilemma or tragedy of the commons situation, both often used to describe grand societal challenges such as overfishing, antibiotic resistance, and climate change.

**Table 1:** Selection of facilitators generating and sustaining successful collective action

Facilitator	Function	References
Intra-actor facilitators		
Pro-social preferences/Values/Personal norms and beliefs	Increasing concern for other actors’ needs and preferences, increase the likelihood of cooperative behaviour	Kerr (1995), Fehr and Schmidt (1999), Fehr and Gächter (2002), and Bogaert et al. (2008)
Fairness	Perception of procedural and distributional fairness affects actors’ propensity to cooperate	Wilke (1991), Sutinen and Kuperan (1999), and Tyler (2010)
Inter-actor facilitators		
Trust	If an actor relies on other actors propensity to cooperate, then cooperation increases	Levi and Stoker (2000), Uslaner (2002), Ostrom and Walker (2003), Cook et al. (2005), Nannestad (2008), Krueger et al. (2017), and Van Lange et al. (2017)
Reciprocity	Other actors previous action affect the propensity to cooperate	Fehr and Gächter (2000b), Fischbacher et al. (2001) and Ostrom and Walker (2003)
Conditional cooperation	If other actors cooperate, then the likelihood of cooperation increases	Levi (1998) Gächter and Herrmann (2009), and Chaudhuri (2011)
Communication	Communication facilitates coordination and information exchange between actors	Dawes et al. (1977), Sally (1995), Dietz et al. (2002), and Balliet (2010)
Power	Veto player, power asymmetries, and other heterogeneities affect actors’ propensity to cooperate.	Baland and Platteau (1996), Varughese and Ostrom (2001), Kopelman et al. (2002), Tsebelis (2002), and Poteete and Ostrom (2004)
Punishment	Sanctioning of non-cooperative behaviour increases the likelihood of cooperation	Fehr and Gächter (2000a), Balliet et al. (2011), and Balliet and Van Lange (2013)
Societal facilitators		
Social norms	Societal (descriptive and prescriptive) norms affecting single actors’ propensity to cooperate	Ostrom (1998), Stern et al. (1999), and Biel and Thøgersen (2007)
Local institutions	Sound institutional design supporting observability, monitoring and sanctioning increase the likelihood of cooperative behaviour	Baland and Platteau (1996), Varughese and Ostrom (2001), Kopelman et al. (2002), Tsebelis (2002), and Poteete and Ostrom (2004)
Technology	Technological solutions increase the propensity for cooperation primarily by reinforcing and supporting other facilitators	Ostrom (2000) and Agrawal (2001)

The premise also highlights the need for complementary mechanisms and institutions that can generate and sustain larger-scale collective action, as well as support actors in overcoming the cooperation and coordination problems that they face. Thus, given that a certain large-scale collective action problem is characterised by a large number of actors, spatial distance, temporal distance, and complexity, and furthermore that these characteristics are generating stressors that counteract collective action, a second premise can be established:

The larger the scale of the collective action problem, the smaller the likelihood that spontaneous collective action will emerge and be sustained.

This second premise is very much in line with seminal works on collective action, such as Olson (1965, p. 2), which argues that “unless the number of individuals in a group is quite small, or unless there is coercion or some other special device to make individuals act in their common interest, rational, self-interested individuals will not act to achieve their common or group interests”.^1^ It is also very much in line with Ostrom (1998, p. 1), who argues that solving large-scale collective action problems is “the core justification of the state”, also backed up by the assertion by Mansbridge (2014, p. 10) that overcoming large-scale collective action problems is “the most significant reason for government”. Based on this, we claim that:

The larger the scale of the collective action problem, the more likely it is that collective action will have to be generated through (third-party) interventions.

Hence, we argue for interventions that could break or weaken some of the collective action stressors or reinforce collective action facilitators, thereby leading to successful larger-scale collective action despite the characteristics described above. We use “third-party intervention” as a generic term to describe situations in which a party that is external to the collective action problem increases the likelihood of collective action in a controlled and managed way (see Ensminger 1996; Greif 2006; Mansbridge 2014). These interventions may or may not be coercive, depending on a number of factors, including ideology, political institutions, history, and culture. In this endeavour, we are particularly inspired by Ostrom’s polycentric framework (Ostrom 2010), thereby defining such a third party as either a formal or informal institution with the capacity (and legitimacy) to affect either the stressors or the facilitators, or both. Examples of such third parties include states, city governments, regional authorities, community leaders, and even businesses, trade associations, and in some cases multilateral organisations. For example, the Intergovernmental Panel on Climate Change (IPCC) is a multilateral organisation that targets the complexity of the global climate change problem in an attempt to facilitate agreements between countries; however, it lacks enforcement mechanisms. The World Trade Organization is also a multilateral organisation, but unlike the IPCC, it has the power to sanction non-compliance (although the effectiveness of sanctioning measures depends on the relative economic power of the states concerned). Whether an international treaty, such a multilateral environmental agreement (MEA) can be regarded as a “third party” in relation to the states that are parties to it, is a complex issue that needs to be assessed in the light of the features and mechanisms of each specific agreement and is beyond the scope of this article.

Importantly, in most cases, any third-party intervention will require a certain amount of legitimacy and acceptability among involved actors to be effective in generating collective action and avoiding free-rider behaviour. In addition, the creation or evolution of a third party is, of course, a non-trivial issue. That is, while a third party has the potential to generate collective action, the very creation and maintenance of such a third party pose a collective action problem in itself. This so-called second-order dilemma stems from the fact that even if every actor would benefit from a third party solving collective action problems, this benefit would potentially accrue even if every single actor did not contribute to the existence or sustenance of the third party. Hence, there is an incentive to free ride, not only in respect to the collective good itself, but also in respect to the creation and maintenance of the third party facilitating the creation of a collective good (Becker and Ostrom 1995; Heckathorn 1996). Moreover, a third party may very well be an integral part of producing a collective action problem among individual actors. That is, while some third parties are extremely effective in fostering collective action, others display substantial shortcomings in terms of willingness or capacity to produce collective goods. For example, in many developing countries the state tends not to be perceived of as a vehicle for collective action but rather as a resource to be appropriated in order to fulfil short-term particularistic objectives (Englebert 2000). Thus, the role of the third party—and its relationship to the actors that are to be governed—needs to be problematised and studied further in future research.

**Fig. 1:**
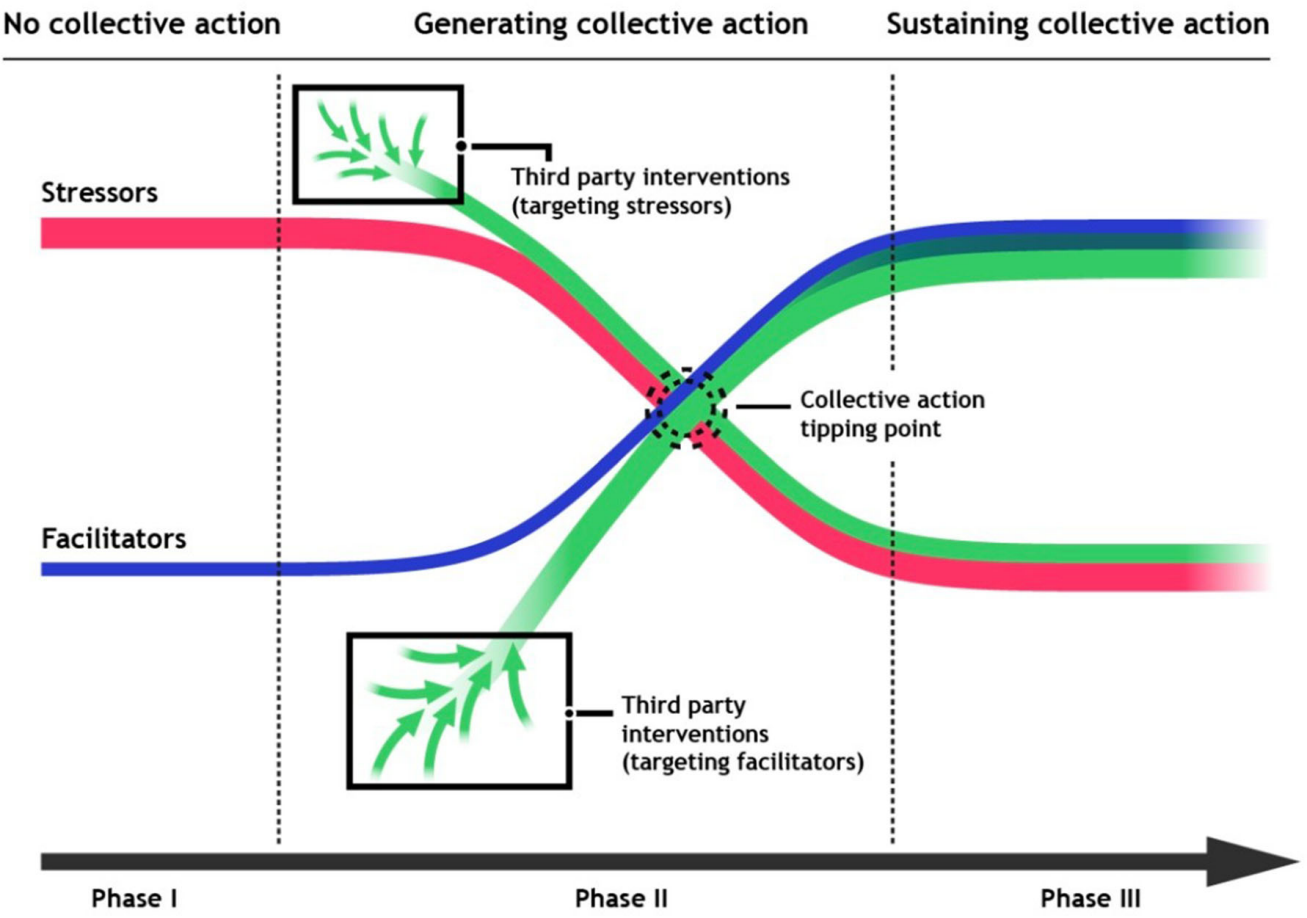
Generating and sustaining large-scale collective action

Using a graphical illustration of our framework (Fig. 1), we summarise how a third-party intervention of any sort should aim at decreasing the stressors or increasing the facilitators so that the propensity for actors to engage in collective action is increased and sustained over time.

Phase I constitutes a situation with no collective action and where stressors such as anonymity, uncertainty, and heterogeneity outweigh collective action facilitators. This creates a demand for a third party. In Phase II, third-party interventions are introduced with the aim of weakening stressors or supporting facilitators, or both, and potentially also altering the character of the large-scale collective action problem (for example, by introducing legal and market-based instruments such as property rights or prices) and turning it into a free-rider problem, which is typically more manageable. Ideally, this leads to a *collective action tipping point*. Phase III constitutes a stable situation where facilitators in combination with third-party interventions outweigh the impact of stressors, sustaining collective action.

In Phase I, the large-scale characteristics of the collective action problem activate a number of stressors, such as anonymity, uncertainty, and heterogeneity, which have negative impacts on various mechanisms that tend to generate or facilitate collective action. Given our premise on large-scale collective action in this situation, there likely will not be any spontaneous collective action.

In Phase II, large-scale collective action is enabled by the introduction of interventions. However, for *successful* collective action to be generated, a *collective action tipping point* needs to be reached. This refers to the point beyond which collective action is sufficient to overcome the problem. This could be when overfished fish stocks recover or the CO_2_ levels in the atmosphere begin to stabilise.

While the overarching aim of the interventions is to manage the large-scale collective action problem, these interventions can function in several ways. First, interventions can work by reducing stressors, supporting facilitators, or both. The presence of a third party can reduce stressors such as anonymity and uncertainty in actor interactions, such as through monitoring and surveillance or a reporting system. It can also promote and foster pro-social preferences, cooperative social norms, values, and trust among the involved actors. Finally, the *lack* of a facilitator can also constitute a collective action stressor. For example, lack of inter-personal trust (and even more so *dis*trust) can effectively counteract cooperation. In this case, a trust-building intervention may strengthen this facilitator and, thus, simultaneously weaken the stressor.

Second, interventions can be used to more directly change actors’ behaviour, such as by altering the character of the large-scale collective action problem. Examples of such interventions are legal and market-based instruments, behavioural interventions, and more direct interventions such as incentive-based policy instruments, command and control, and regulatory and facilitating measures such as technological standards and subsidies. However, third-party interventions can also focus explicitly on removing existing bad rules or other institutional barriers to collective action, such as harmonising legislation or other institutions with conflicting instructions and goals.

Third, the two different types of interventions can work in tandem. One such example may be a carbon or chemical tax that is implemented to correct for a market failure (an externality), thereby changing the characteristics of the coordination situation. This is the direct effect of the intervention. However, one can hypothesise that certain facilitators could also be affected by such a carbon tax through alignment of motivational concerns for individuals. For example, if an individual holds a norm not to emit carbon, but carbon is not properly priced, budget concerns may override the social norm as a motivational concern.

It is also important to emphasise that interventions can originate from third parties other than the government. One example is organisations working with eco-labelling. The intervention (labelling) may generate collective action by targeting one or several stressors (e.g. decreasing uncertainty by increasing knowledge among resource users).

*To sustain* collective action (Phase III), i.e. avoid a situation collective action is *reverted* to non-collective action, it is required that the facilitators together with the interventions continuously outweigh the stressors. However, various possible developments can arise whereby the strengths of the stressors could increase, stay constant, or diminish, which in turn will determine the actual need for sustaining or increasing the facilitating factors or implementing new interventions for successful collective action over time. Alternatively, society may adapt to the interventions such that the need for continued interventions gradually wanes. An example of the latter case is that once the automobile market has fully shifted from being fossil fuel-based to being electricity-based, or at least based on renewable energy with all necessary infrastructure put in place, it is likely that there will not be a shift back to a market based on fossil fuels, even if the active interventions generating and sustaining this shift are being removed.

## Concluding remarks

*Large*-*scale* collective action problems are at the heart of humanity’s most pressing challenges, including natural resource depletion, antibiotic resistance, migration, and climate change. The phenomenon of collective action has been extensively and systematically studied in the social sciences, through game-theoretical exercises, lab experiments, and numerous case studies, where the typical definition of a collective action problem has been a *dilemma* situation wherein the actors’ short-term self-interest is inconsistent with longer-term collective interests, thus generating a substantial risk that the collective benefit is not produced at all. In this paper, we propose a wider definition including other *coordination problems* that also require collective action to be overcome. In particular, there are major differences between small-scale and large-scale collective action, and furthermore, every large-scale collective action problem is more or less unique and should, therefore, be analysed accordingly.

We have defined and described the main characteristics of a large-scale collective action problem to show the great variation possible among different problems. Further, we have outlined how these characteristics generate more or larger stressors, hampering voluntary, and especially spontaneous, collective action, the larger the scale of the collective action problem. Thus, for large-scale collective action to be generated and lead to a collective action tipping point, this problematic relationship among scale, core characteristics, stressors, and cooperative behaviour requires different forms of interventions by external (third) parties. To show the different phases involved in achieving long-lasting collective action, going from no cooperation, to generating and eventually sustaining cooperation, we presented a graphical illustration of our analytical framework capturing the connection between third-party interventions and large-scale collective action.

Our framework has several pedagogical and scientific merits. First, the identification of scale and the division of collective action characteristics and stressors and their relationships enhance our understanding of why there is so little spontaneous and self-organised collective action regarding large-scale collective action problems, even though the scholarly community is doing its best to communicate and inform about the current state of the many existing challenges. The answer is simply that in the case of most large-scale collective action problems, there are a large number of stressors hampering any individual actor’s willingness to spontaneously start cooperating (regardless of what others do).

Second, the systematic identification of stressors helps us understand and explain why many large-scale collective action problems are not proper social dilemmas, i.e. that all defecting resource users would, ‘sit in the same boat’, so to speak, constantly and equally risking the loss of their joint resource unless they start cooperating. As we have seen, for many large-scale collective action problems, this situation is far from true. However, what seems to be true for all large-scale collective action problems is that for collective action to be successful, certain actors must act against their own short-term self-interest, or certain agents must act against the short-term interest of their principals.

Third, by treating all collective action problems as strict social dilemmas (Ostrom 1998) or tragedy of the commons problems (Hardin 1968), policy-makers and the scholarly community risk missing the potential or underestimating the importance of developing collective action interventions and policies—that is, to paraphrase Abraham Maslow (1966): they may tend to see all problems as nails, because the only tool they have access to is a hammer. The framework introduced in this paper can enrich scholars’ policy and analytical toolbox used to map out the key actors and stressors associated with each unique larger-scale collective action problem. Based on that information, scholars, and ultimately also policy-makers, can then systematically analyse and elaborate on what would be effective third-party interventions in order to solve the problem—i.e. designing and implementing policies and policy instruments that effectively carry out the behavioural changes necessary for the overall problem to be overcome. The success for each such third party (i.e. to ensure actors’ compliance with interventions) is determined by a number of factors, both related to the third party itself (e.g. type of political system, quality of government) but also individual-level factors linked to the actors who are supposed to carry out the behavioural changes. This is, however, a topic of its own and we welcome research that systematically addresses what interventions and policy instruments that are most suitable, depending on how every unique third party is constituted.

Finally, as a reader one might by now ask if there are any good examples of successful large-scale collective action, and if so, what the framework presented in this paper can say about what generated that collective action? The Montreal Protocol on Substances that Deplete the Ozone Layer can serve as a useful example since it is often viewed as a highly successful form of international cooperation (Benedict 1989; Morrisette 1991; Sunstein 2007). While at a first glance, the Montreal Protocol seems to effectively tackle a large collective action problem that scores high, both on number of actors involved (states, industry and individual actors on a global scale), spatial and temporal distance, and probably complexity as well, a closer investigation reveals numerous factors that ameliorate the characteristics and stressors linked to them. Among these are a tangible and easy to communicate problem (e.g. elevated risk for skin cancer); strong scientific consensus regarding its causes and solutions; availability of reasonable substitutes for ozone-depleting CFCs; and benefits of taking action that were calculated to outweigh costs for most countries and even supported unilateral action by influential states (Sunstein 2007). Although, for example, the protocol’s prohibitions on importing and exporting many ozone-depleting substances and products worked to further reduce complexity (by preventing industries from relocating to non-parties while still having access to the markets of the parties), there is much to suggest that important stressors were comparatively modest, making ozone layer depletion a less challenging large-scale collective action problem to begin with and thus one that was not too challenging to address. For example, this can be compared to climate change, the characteristics of which give rise to very potent stressors, with consequences that are less easy to predict, a scientific basis that is constantly challenged and hard to explain (e.g. difference between weather and climate and constantly evolving models that can seem contradictory), starkly differing cost–benefit analysis between countries and companies, and the lack of a single solution to the problem, just to mention some obvious differences.

The two examples illustrate what we have tried to explain and highlight in this perspective article: that even if states (a typical category of third parties) may manage to come to an agreement on how to cope with a global challenge, such as emissions of CFCs or CO_2_, only half the battle is won. The next challenge is then for each third party to design and implement policies and policy instruments that effectively make their respective consumers and producers carrying out the behavioural changes necessary for the overall problem to be overcome (and to which each actor only contributes a minimum). In addition, the above examples of the Montreal Protocol and climate change show the importance of facilitators that can reduce the potency of collective action stressors.

Summing up, future research should apply the suggested framework on different large-scale collective action problems in order to better understand how the unique nature of every individual large(r)-scale collective action problem generates unique stressors that need to be addressed and approached for the problem to be overcome. It should also focus on investigating which blend, sequence, and pacing of third-party interventions would most successfully generate and sustain collective action in these different situations. In addition, future studies should involve additional actors other than, primarily, individuals.

## Footnotes

1 Simultaneously, it should be noted that although Olson’s claim is reasonably valid in most cases, one cannot fully *exclude* situations where one or a few actors—out of many—may be so powerful or influential that if these relatively few actors voluntarily decide to change from a defective to a cooperative behaviour, this will either be enough to overcome the collective action problem per se, due to these actors’ total impact on a scarce resource (Hardin 2015), and/or this shift towards a cooperative behaviour might even encourage other involved actors to change their behaviour too. Thus, it is not necessarily only the size of the group that matters. Also, the ratio between benefits and costs can be important. To quote Hardin (2015) “If that ratio is very large, then a relatively small fraction of the whole group would already stand to benefit, even if that fractional subgroup alone paid the full cost of the group good.” (Hardin 2015, pp. 40–41). This way of reasoning could for example be used to argue that much would be won for the global collective, in terms of combating global climate change, if only one or a few major emitters of CO_2_ (i.e. US and China) would take a significant step in reducing their emissions.
